# The Quixote project: Collaborative and Open Quantum Chemistry data management in the Internet age

**DOI:** 10.1186/1758-2946-3-38

**Published:** 2011-10-14

**Authors:** Sam Adams, Pablo de Castro, Pablo Echenique, Jorge Estrada, Marcus D Hanwell, Peter Murray-Rust, Paul Sherwood, Jens Thomas, Joe Townsend

**Affiliations:** 1 Unilever Centre for Molecular Science Informatics, Department of Chemistry, Lensfield Road, Cambridge CB2 1EW, UK; 2 SONEX Workgroup for Scholarly Output Notification and Exchange, http://sonexworkgroup.blogspot.com; 3 Instituto de Química Física "Rocasolano", CSIC, Serrano 119, E-28006 Madrid, Spain; 4 Instituto de Biocomputación y Física de Sistemas Complejos (BIFI), Universidad de Zaragoza, Mariano Esquillor s/n, Edificio I+D, E-50018 Zaragoza, Spain; 5 Departamento de Física Teórica, Universidad de Zaragoza, Pedro Cerbuna 12, E-50009 Zaragoza, Spain; 6 Departamento de Bioquímica y Biología Molecular y Celular, Universidad de Zaragoza, Pedro Cerbuna 12, E-50009 Zaragoza, Spain; 7 Kitware, Inc., 28 Corporate Drive, Clifton Park, NY 12065, USA; 8 STFC Daresbury Laboratory, Daresbury Science and Innovation Campus, Warrington WA4 4AD, UK

## Abstract

Computational Quantum Chemistry has developed into a powerful, efficient, reliable and increasingly routine tool for exploring the structure and properties of small to medium sized molecules. Many thousands of calculations are performed every day, some offering results which approach experimental accuracy. However, in contrast to other disciplines, such as crystallography, or bioinformatics, where standard formats and well-known, unified databases exist, this QC data is generally destined to remain locally held in files which are not designed to be machine-readable. Only a very small subset of these results will become accessible to the wider community through publication.

In this paper we describe how the Quixote Project is developing the infrastructure required to convert output from a number of different molecular quantum chemistry packages to a common semantically rich, machine-readable format and to build respositories of QC results. Such an infrastructure offers benefits at many levels. The standardised representation of the results will facilitate software interoperability, for example making it easier for analysis tools to take data from different QC packages, and will also help with archival and deposition of results. The repository infrastructure, which is lightweight and built using Open software components, can be implemented at individual researcher, project, organisation or community level, offering the exciting possibility that in future many of these QC results can be made publically available, to be searched and interpreted just as crystallography and bioinformatics results are today.

Although we believe that quantum chemists will appreciate the contribution the Quixote infrastructure can make to the organisation and and exchange of their results, we anticipate that greater rewards will come from enabling their results to be consumed by a wider community. As the respositories grow they will become a valuable source of chemical data for use by other disciplines in both research and education.

The Quixote project is unconventional in that the infrastructure is being implemented in advance of a full definition of the data model which will eventually underpin it. We believe that a working system which offers real value to researchers based on tools and shared, searchable repositories will encourage early participation from a broader community, including both producers and consumers of data. In the early stages, searching and indexing can be performed on the chemical subject of the calculations, and well defined calculation meta-data. The process of defining more specific quantum chemical definitions, adding them to dictionaries and extracting them consistently from the results of the various software packages can then proceed in an incremental manner, adding additional value at each stage.

Not only will these results help to change the data management model in the field of Quantum Chemistry, but the methodology can be applied to other pressing problems related to data in computational and experimental science.

## Background

### Quantum Chemical calculations and data

High-level quantum chemical (QC) methods have become increasingly available to the broader scientific community through a number of software packages such as Gaussian [1], GAMESS(US) [2], GAMESS-UK [3], NWChem [4], MOLCAS [5] and many more. Additionally, the cost of computer power has experienced an exponential reduction in recent decades and, more importantly, sophisticated approximations have been developed that pursue (and promisingly approach) the holy grail of linear scaling methods [6,7]. This has enabled any researcher, with no specific QC training, to perform calculations on large, interesting systems using very accurate methods, thus generating a large amount of valuable and expensive data. Despite the scientific interest of this data and its potential utility to other groups, its lack of homogeneity, organization and accessibility has been recognized as a significant problem by important agents within the scientific community [8,9].

These problems, and specially the ones related to the accessibility of data have many consequences that reduce the efficiency of the field. As mentioned, QC methods are computationally expensive: the scaling of the computer effort and storage of high-level computations with the size of the system (*N*) is harsh, reaching, for example, *N*^7^, for the most expensive and most accurate wavefunction-based methods, such as Coupled Cluster [10-12]. This makes it very difficult for groups that cannot use supercomputing facilities to have access to high-quality results, even if they possess the expertise to analyze and use the data. Even groups that do have access to powerful computational resources, given the lack of access to previously computed data by other researchers, often face the choice between *two inefficient *options: either they spend a lot of human time digging in the literature and contacting colleagues to find out what has already been calculated, or they spend a lot of computer effort (and also human time) calculating the needed data themselves, with the risk of needlessly duplicating work.

Another problem originating in the lack of access to computed QC data and the very large number of methods available, is that users typically do not have the integrated information about which method presents the best accuracy *vs*. cost relation for a given application. The reason is that comparing one quantum chemical method with another, with classical force fields or with experimental data is non-trivial, the answer frequently depending on the studied molecular system and on the physical observable sought. Moreover, all the details and parameters that define what John Pople termed a *model chemistry *[13], *i.e*., the exact set of rules needed to perform a given calculation do not obey a continuous monotonic function. Thus increasing the expense and "accuracy" of a calculation may not always converge to the "correct" solution. As a consequence, the quality of the results does not steadily grow with the computational effort invested, but rather there exist certain tradeoffs that render the relation between them more involved [14-16]. Hence, not only the choice of the more efficient QC method for a given problem among the already existing ones, but also the design of novel model chemistries becomes 'more an art than a science' [17], based more on know-how and empiricism than in a set of systematic procedures.

### Design of Scientific data repositories

In this paper we describe a novel, flexible, multipurpose repository technology. It arises out of a series of meetings and projects in the computational chemistry (compchem) community which have addressed the desire and need to have repositories available for capturing and disseminating the results of QC calculations. It is also strongly influenced by the eScience ("cyberinfrastructure", "eResearch") programs which have streesed the value of instant semantic access to research information from many disciplines, and by the Open Innovation vision supported by the Scientific Software Working Group of CECAM (Centre Européen de Calcul Atomique et Moléculaire) http://www.cecam.org/, which seeks an innovation model based on sharing, trust and collaboration, and which recognizes the important role played by the availability of reference data and archives of outputs of calculations and simulations. It also coincides with the increasing mandates for data publication from a wide range of funders; our repository can address a large part of these requirements.

This paper describes a distributed repository technology and the social aspects associated with developing its use. The technology is robust and deployed but the way it may be used is at a very early stage. We address known social issues (sustainability, quality, etc.) but expect that deployment, even in the short term, may look very different from what is reported.

The development and acceptance of Wikipedia may act as a valuable guide and it represents a community-driven activity with community-controlled quality. Although variable, we believe that articles for most mainstream physical sciences are reliable. Thus to help understand and represent moments of inertia in computational chemistry we can link to Wikipedia http://en.wikipedia.org/wiki/Moment_of_inertia. This contains many hundreds of edits over eight years from many authors - it is almost certainly "correct". Quixote has many of the same features - anyone can contribute content and repurpose it. We expect a culture to emerge where the community sets guidelines for contributions and corrections/annotations. We are building filters ("lenses") so that the community can identify subcollections of specific quality or value.

The background to Quixote includes a number of meetings and projects which specifically addressed the development of infrastructure in computational chemistry and materials. The goal of these was to explore the commonality between approaches and see how data and processes could interoperate. One (Materials Grid) also addressed the design and implementation of a repository for results.

• 2004: A meeting under the UK eScience program "Toward a common data and command representation for quantum chemistry" http://www.nesc.ac.uk/action/esi/contribution.cfm?Title=394.

• 2006: A meeting under the auspices of CECAM "Data representation and code interoperability for computational materials physics and chemistry" http://www.cecam.org/workshop-50.html

• 2005-2010: A 5-year project under the COST D37 program to develop various aspects of interoperability both within the calculation (Q5COST) and between programs (WG5).

• A funded project in computational materials ("Materials Grid") http://www.materialsgrid.org/ which resulted in considerable development of CML specifications and trial implementations in a number of codes (CASTEP, DLPOLY).

These meetings and projects were exploratory and localized. Within them there was a general agreement that interoperability and access to results would be a great benefit. But they also highlighted the problem that infrastructure development is expensive and, if public, requires political justification for funding. Such funding is perhaps most likely to come from supranational efforts such as computational Grids, where there is a clear imperative for making services as accessible as possible. In COST-D37 the funding was for meetings and interchange visits; the WG5 community made useful but limited progress without dedicated developer or scientist funding.

There is often a vicious circle here - a frequent reason for not adopting a new technology in chemistry is "there is no demand for it". This becomes a self-fulfilling prophecy and naturally limits innovation. It is also true that people are often only convinced by seeing a "working system" - hypothetical linkages and implementations have often been wildly optimistic. Therefore without seeing a working repository it is difficult to know what its value is, or the costs of sustaining it.

However the Internet age shows that it is much easier, cheaper and quicker to get new applications off the ground. It should be possible, in a short time and with modest effort, to create a system which demonstrates semantic interoperability and to convince a community of its value. We have successful examples of this reported elsewhere in this issue (OSCAR, Open Bibliography) where an early system has caught the imagination and approval of a section of the community.

### Existing related projects

These issues, and undoubtedly more that will appear in the future, together with a wealth of scientific problems in neighbouring fields, could be tackled by public, comprehensive, up-to-date, organized, on-line repositories of computational QC data. Additionally, several fields reporting experimental data require it to be presented in a standard validatable form. The crystallography community has long required deposition of data as a prerequisite for publication, and this is now enhanced by machine validation (the CheckCIF philosophy and program http://checkcif.iucr.org/). When data are submitted, the system can comment on whether all appropriate data are present, inspect their values and compare either with known ranges or re-compute relationships between them based on accepted theoretical principles. In this way reviewers and readers can expect that a very large number of potential errors in experiment and publication have been eliminated.

This requirement for deposition of data as part of the publication process is increasingly common in bioscience, like genetics or proteomics, where the NCBI GenBank http://www.ncbi.nlm.nih.gov/genbank/ or the Protein Data Bank (PDB) http://www.rcsb.org/pdb/home/home.do constitute very successful examples of data sharing and organization. In an age in which both the monetary cost and the accuracy of QC calculations rival those of experimental studies, the need to extrapolate the model to this field seems obvious. We also note that funders are requiring that data be deposited as part of the condition of funding. On the one hand, there exist some in-house solutions that individual research groups or firms have built in order to implement a local-scale data management solution. This is the case of David Feller's Computational Results Database http://tyr3.chem.wsu.edu/~feller/Site/Database.html[18], an intra-lab database to store and organize more than 100,000 calculations on small to medium-sized molecules, with an emphasis on very high levels of the theory. Also, the commercial standalone application SEURAT http://www.synapticscience.com/seurat/ can open and parse QC data files and allows for metadata customization by the user, thus providing some limited, local databasing capabilities. In the same family of solutions, ChemDataBase [19] is a data management infrastructure mainly focused on virtual screening which presents the distinctive feature of being able to create and retrieve databases over grid infrastructures. Packages for interacting with QC codes (launching, retrieving and analyzing calculations), such as ECCE http://ecce.emsl.pnl.gov/index.shtml or Ampac http://www.semichem.com/ampac/afeatures.php, have modest data management capabilities too, although only insofar as it helps to perform their main tasks, and they can be regarded as intra-lab solutions as well. Probably the most complete in-house infrastructure of which we are aware of is the RC^3 ^(Regional Computational Chemistry Collaboratory) developed by the group of David Dixon at the Department of Chemistry of the University of Alabama. The main objective of RC^3 ^is to perform the everyday data backup, collection and metadata assignment for calculations, and to organize them for research purposes. At the time of writing, RC^3 ^has been tested by 36 users for more than a year, and backed-up and organized 1.6 million files, amounting to 1.5TB of data storage. The database contains 144,000 records and it can currently parse multiple QC data formats.

### Heterogeneous data repositories

A different category of data management solutions from the one discussed above is that constituted by a number of online web-based repositories of QC calculations, normally developed by one research group with a very specific scientific objective in mind. Among them, we can mention the NIST Computational Chemistry Comparison and Benchmark DataBase (CCCDB) http://cccbdb.nist.gov/, which contains a collection of experimental and calculated *ab initio *thermochemical, vibrational, geometric and electrostatic data for a set of gas-phase atoms and small molecules; the Benchmark Energy and Geometry DataBase (BEGDB) www.begdb.com [20], which includes geometry and energy CCSD(T)/CBS calculations as well as other high-level calculations, with a special emphasis on intermolecular interactions; the DFT Database for RNA Catalysis (QCRNA) http://theory.rutgers.edu/QCRNA/[21], which contains high-level density-functional electronic structure calculations of molecules, complexes and reactions relevant to RNA catalysis; the Atomic Reference Data for Electronic Structure Calculations http://www.nist.gov/pml/data/dftdata/index.cfm[22] compiled at NIST, containing total energies and orbital eigenvalues for the atoms hydrogen through uranium, as computed in several standard variants of density-functional theory, or the thermochemistry database at the Computational Modeling Group of Cambridge's Department of Chemical Engineering http://como.cheng.cam.ac.uk/index.php?Page=cmcc, collecting thermochemical data of small molecules, powered by RDF and SPARQL and offering the output files of the calculations, together with the parsed CML http://cml.sourceforge.net[23].

Apart from these solutions (either local or web-based), in which one or a few groups build a complete data management infrastructure, one can also consider the possibility of adopting a modular approach, in which different researchers tackle different parts of the problem, whilst always enforcing the maximum possible interoperability between the modules. The Blue Obelisk group http://www.blueobelisk.org/[24] has been championing this approach for a number of years now, and many of the developers of the tools discussed below are members of it. In this category of solutions, we can also mention the Basis Set Exchange (BSE) https://bse.pnl.gov/bse/portal[18,25], which provides an exhaustive list and definition of the most common basis sets used in QC calculations, thus facilitating the definition and implementation of semantic content regarding the method used, as well as improving the interoperability among codes at the level of the input data; modern tagging and markup technologies like XML and RDF together with the building of semantic dictionaries, not only to promote interoperability, but to do it in a web-friendly manner that allows one to easily plug modules and build complex online data management projects; the CML language (a chemical extension of XML) [23] is also one of the few cases in which a common semantics has been widely adopted by the chemistry community, and its extension to the QC field is one of the cornerstones of the Quixote project described here. Also on the interoperability front, we can mention the cclib http://cclib.sf.net[26] and CDK http://cdk.sf.net[27] libraries, as well as the OpenBabel toolbox http://openbabel.org, which provide many capabilities for reading, converting and displaying QC data in many formats. Regarding the ease of use of possible data management solutions, the Open Source molecular editor and visualizer Avogadro http://avogadro.openmolecules.net can certainly be used as a useful module in complex projects, and in fact the design of Quixote is being carried out in collaboration with the developers of Avogadro, with the intention of efficiently interfacing it in future versions. The Java-based viewer Jmol http://jmol.sourceforge.net/ performs similar tasks.

All in all, and despite the numerous efforts described above, it is clear that a global, unified, powerful solution to the management of data in QC does not exist at present; at the same time that the new internet-based technologies, the existence of vibrant communities, and the wide availability of powerful software to perform the calculations, and to convert and analyze the results, all seem to indicate that the field is ripe to produce a revolutionary (and much needed) change in the model. In this article, we present the beginnings of an attempt to do so.

### The Quixote solution

The catalyst for Quixote was a meeting on interoperability and repositories in QC held at ZCAM (Zaragoza Scientific Center for Advanced Modeling), Zaragoza (Spain) in September 2010. There was general agreement on the need for collection and re-dissemination of data. In the final discussion a number of participants felt that there was now enough impetus and technology that something could and should be done. This wasn't a universal view, and we are aware that Quixote is unconventional in its genesis and aspirations - hence the name, reflecting a difficult but hopefully not impossible dream.

We decide to pursue this as an informal "unsponsored" project. It is not actually "unfunded", in that we recognize the critical and valuable cash and in-kind support of several bodies, including CECAM, STFC Daresbury Laboratory, EPSRC, JISC, ZCAM, and the employers of many of the participants. In particular we have been able to hold, and continue to hold, meetings. But there are no sponsor-led targets or requirements. In this it has many of the features of successful virtual projects in ICT (such as Apache, Linux, *etc*.) and communal activities such as Wikipedia and Open Street Map.

Speed and ambition were critical and project management has been by deadlines - external events fixed in time for which the project had to have something to show. These have included:

• An *ad hoc *meeting in 2010-10 in Cambridge where a number of the participants happened to be. This was to convince ourselves that the project was feasible in our eyes

• The PMR symposium 2011-01 that has catalysed this set of articles

• A workshop 2011-03 at STFC Daresbury Laboratory to demonstrate the prototype to a representative set of QC scientists and code developers

• Open repositories (OR11) 2011-06 where the technology was presented to the academic repository community as an argument for the need for domain repositories

• (planned) A meeting in Zaragoza 2011-08 where the argument for domain repositories will be demonstrated by Quixote.

As of 2011-06 we have a working repository with over 6000 entries, which are searchable chemically, by numeric properties and through metadata.

**Figure 1:**
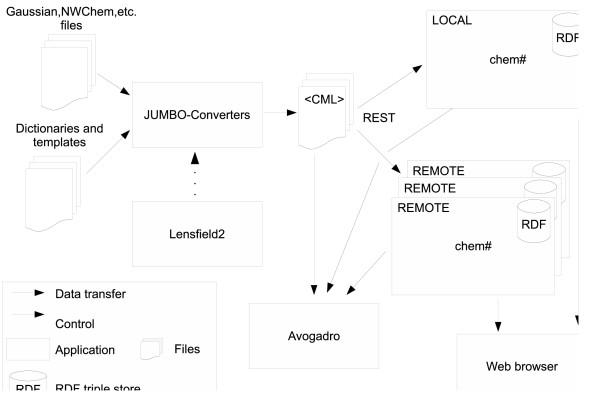
**Quixote architecture and conversion workflow**. The user instructs Lensfield2 to convert output files of different computational chemistry codes into semantically rich CML files. The conversion is performed by JUMBO-Converters following the hints provided in the dictionaries and templates. The generated CML files are then transferred to one or more local and remote chem# repositories using a RESTful web API. The user can search and browse those repositories with a web browser, and can also manipulate and visualize the CML files with Avogadro.

**Figure 2:**
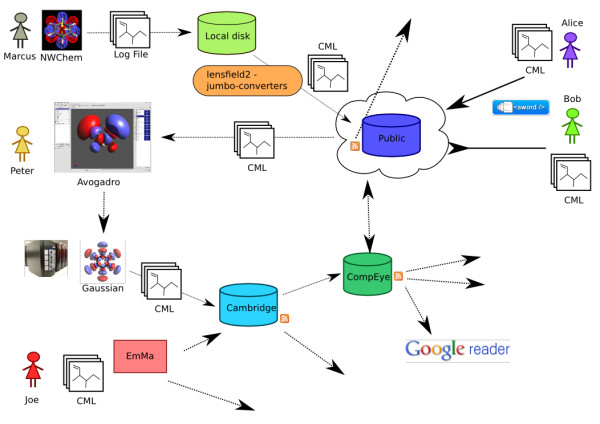
**Quixote distributed repositories**. A schematic view of distributed Quixote repositories. Some repositories push documents to the public web, others aggregate from it. There is (deliberately) no check on whether repositories have identical documents. Users can build search strategies that look for individual entries with specific data or make collections of documents that share or contrast properties.

Our primary goal has been to build working, flexible technology without being driven by specific use-cases. This can be seen as heresy, and indeed we might regard it as such ourselves, if it were not that we have spent about 10 years working in semantic chemistry, computational chemistry and repositories and so have anticipated many of the possible use cases and caveats. To help show Quixote's flexibility we now list a number of use cases, any one of which may serve to convince the reader that Quixote has something to offer:

The Quixote system (Figure 1 shows the workflow, Figure 2 shows the distributed heterogeneity) is very flexible in that it can be installed in several different ways. Here we give a number of possible uses of the system, some of which we have deployed and several more we expect to be useful.

• Collection of results within a group or laboratory. There is a growing desire to capture scientific results at the time of creation, and we have been involved in several projects (CLaRION, JISC XYZ) the impetus of which is to see whether scientists can capture their data as they create it. Computational chemistry is one of the simplest types of results and Quixote has been designed so that a single log file provides most of the input to the repository. This system allows groups and individual researchers to "pick up their results" and transport them to different environments.

• Formal publication in journals and theses. Results in a Quixote repository can be made available to other people and parties in the publication process. For example an author could make their results available to a journal before review so that the editors and reviewers could use the data to assess the value of the science. Similarly a graduate student could make their results available as part of their thesis submission and these could be assessed by the examiners. If the thesis and accompanying data are also published in the institutional repository then this provides a simple but very effective way of capturing and preserving the record of scientific experiments.

• Teaching and learning resources. Quixote can collect resources used for teaching and can also be used to provide subsets of research objects which are valuable for teaching and learning. For example in the current set there are 75 calculations on benzene, mainly from Henry Rzepa's laboratory and these have been deposited by students carrying these out as part of their undergraduate work. This resource allows us to compare methods and to get information and experience which may help us do similar calculations.

• A collaborative central repository for a project. An increasing number of projects are distributed over geography and discipline. (The current Quixote project is an example.) A repository allows different people and groups in the project to share a central resource in an analogous manner to the use of Bitbucket and similar repositories for sharing code.

• A set of reference data and molecules. Quixote allows us to search for different parameters used in a given problem (*e.g*. level of theory, number of orbitals, convergence of results, algorithms, *etc*.).

• Validation sets for software and methods. In a similar manner datasets within Quixote can be used by different groups as reference input to compare results from different programs or different approaches.

• Enrichment of data through curation. Quixote is annotatable, so that it is possible for the world community to add their comments to particular entries. If a result is suspect, an annotation can be added. Similarly it is possible to point out related entries highlighting different scientific aspects.

• Building blocks for calculations. It is often valuable to start from an unknown program resource (*e.g*. a molecule whose structure is known and where the calculations are verified) and to modify it slightly for a related calculation, *e.g*. by adding additional atoms or by refining the calculation parameters.

• Combining data from different sources. As Quixote can also store experimental structures such as crystallographic ones, or experimental data such as spectra it is possible to enhance and combine components of the calculation.

• Data-driven science. Now that computational chemistry is relatively cheap and relatively accessible for a very large number of scientists, we foresee that literally millions of processors will be used routinely to calculate theoretical chemistry results. This allows us to carry out data mining from the Quixote repositories with the possibility of discovering new scientific patterns.

• Indexing the web. In a similar way to our indexing of crystallography through CrystalEye http://wwmm.ch.cam.ac.uk/crystaleye/ we anticipate that web crawlers can increasingly discover and retrieve published computational chemistry.

• Developing software tools. Since Quixote represents an abstraction of many codes, developers writing software for computational chemistry will be able to see the type of semantics which are captured and the structure of the document.

### Quality

The collection of the scientific computional record through Quixote could be regarded as an objective process in that each logfile is sufficiently described from the view of repeatability. Any user of Quixote could, if they had access to the code(s), re-run the calculation and "get the same output". The examples of student calculations on benzene in the current content illustrate this view.

On the other hand it can be objected that unless a calculation is carried out with professional care then it can not only be meaningless but seriously misleading. Non-experts in QC can obtain these results and can misinterpret them. This is true, but it is a fact of modern Open science - results should be and are available to anyone. Science must evolve social and technical methods to guide people to find the data they want. We can buy a kit and in our garages determine the sequence of a gene or protein without realising the potential experimental errors, or the difficulty of describing the species or strain that it came from. We can buy table-top crystallography sets that will automatically solve the structure of almost all crystalline materials. The results of these experiments are valuable if interpreted correctly and much of the time there is little room for serious error. However we might not realise that one lanthanide might be mistaken for another, that crystals can be twinned, and that certain spacegroups are problematic. Similarly the neophyte may not appreciate the difficulty of getting accurate energies, spin densities, non-bonded interactions, and many more subtleties of computational chemistry. But Pandora's box has been opened and computational chemistry is a commodity open to all. Quixote will help us in making our communal judgments.

There are a few objective concerns about quality. The Quixote system converts legacy computational chemistry (logfiles) into semantic form. Automatic conversion will usually have a small number of errors, but mainly in that fields will not be recognized, rather than corrupted. In the early stages the semantics of some quantities may be misinterpreted (many are often laconic "E = 1.2345" - what exactly is E? and what are the units?) Given the exposure of the system to "many eyes" such problems will be few and should be relatively rapid to remove.

The fuzzier concern is whether Quixote can grow to gain the confidence of the QC and the non-QC community. Computational chemistry has the unique feature that anyone in the world, given the same input, will create the same output. The question is not whether the log file is an accurate record of the calculation but whether the calculation is valuable. It is quite possible to create junk, often unknowingly, and the commonest way is by inputting junk. A typical example is that many chemoinformatics programs can garble hydrogen counts and formal charges. However there are several criteria that the Quixote user and community can apply:

• If the methodology is very standard, then the results are likely to be usable in a similar way to other results using the same method. For example a very common combination of method and basis for organic molecules is B3LYP + 6-31G**. If another group has successfully employed this for a set of molecules similar to the user's it is likely to be a useful starting point. This does not of course absolve the user from critical judgement but it is better than having nowhere to start.

• Automated methods can be used to compare the results of calculations for similar molecules or with varied parameters.

• We particularly encourage collections provided by specified individuals or groups. We have made two available in the current release (Dr. Anna Croft, Prof. Henry Rzepa). The user can browse through collections and get an idea of the type of calculation and the quality of metadata.

• Are the data coupled to publication? In CrystalEye almost all records are coupled to primary publications which can be read by the user (assuming that they have access to the journal). There is no technical barrier why this should not be done for articles and theses in computational chemistry. This is harder in compchem until the community develops a culture of publishing data concurrently with articles.

• Have the entries been annotated? This feature will shortly be available in Quixote, probably through blogging tools.

• Are there criteria for depositing an entry in the particular Quixote repository? Since we expect there to be many repositories, some of them can develop quality criteria for deposition. Some, perhaps the majority, may have human curators. In the first instance it will be important that users can assess the quality of a particular Quixote repository and we are appealing to any scientist who have collections of computational chemistry data that they would be prepared to make available. We expect that there will be a range of levels of quality in Quixote repositories. For example a crawler visiting random web sites for data might store these in an "unvalidated" repository. Users could examine this for new interesting entries and make their own decisions as to their value. The web has many evolved systems for the creation of quality metrics (popularity, usage, recommendations, *etc*.) and many of these would make sense for compchem. A journal might set up their own repository (as is done for crystallography). A department could expose its outputs (and thereby gain metrics and esteem) and the contents would be judged on the assessment of the creators.

## Methods

All materials and methods mentioned here are available as Open Source/Data from the Quixote site or the WWMM Bitbucket repository. A small amount is added as appendixes to guide the reader.

### Concepts and vocabulary

In any communal system requiring interoperability and heterogeneous contributions it is critical to agree concepts and construct the appropriate infrastructure. Chemistry has few formal shared ontologies and Quixote explores the scope and implementation of this for QC.

We draw inspiration from formal systems such as the Crystallographic Information File (CIF) created over many years by the International Union of Crystallography (IUCr). This is a community activity with medium-strong central management - the community has an input but there are formal procedures. It works extremely well and is universally adopted by crystallographers, instrument manufacturers, and publishers. The vocabulary and semantics have been developed over 20 years, are robust and capable of incremental extension. We take this as a very strong exemplar for Quixote and more widely QC.

We believe that almost all QC codes carry out calculations and create outpus which are isomorphic with other codes in the community. Thus an "electric dipole", "heat of formation" or a "wavefunction" is basically the same abstract concept across the field. The values and the representation will be code-dependent but with the appropriate conversions of (say) units, coordinate systems and labelling, it is possible to compare the output of one code with another. This is a primary goal of Quixote, and we work by analysing the inputs and outputs of programs as well as top-down abstractions. It also means that Quixote is primarily concerned with what goes into and comes out of a calculation rather than what is held inside the machine (the data model and the algorithms).

### Community development

From the human resource point of view, the Quixote project operates on a decentralised approach with no central site and with all participants contributing when available, and in whatever quantity they can donate at a particular time. For that reason, different parts of the project progress at variable speeds and technically independently. This means that there is very little effort required in collating and synthesising other than the general ontological problem of agreeing within a community the meaning deployment and use of terms and concepts.

The work is currently driven (*cf*. use cases) by datasets which are available. This drives the need to write parsers, collate labels into dictionaries, and collate results. In the week of 2011-05-09, for example, we ran daily Skype conferences, with Openly editable Etherpads http://quixote.wikispot.org/ generously provided by the Open Knowledge Foundation (OKF) http://okfn.org/. The participants created tutorial material, wiki pages, examples and discussions which over the week focused us to a core set of between 20-50 dictionary entries that should relate to any computational chemistry output. The input to this effort was informed by logfiles from the Gaussian, NWChem, Jaguar and GAMESS-UK programs.

The initial approach has been to parse logfiles with JUMBO-Parser, as this can be applied to any legacy logfiles and does not require alterations of code. (At a later date we shall promote the use of CML-output libraries in major codes.) At this stage it is probably the best approach to analyse the concepts and their structure. A JUMBO-Parser is written for each code and run over a series of example logfiles. Ideally every part of every line is analysed and the semantic content extracted. In practice each new logfile instance can bring novel structure and syntax but it is straightforward to determine which sections have been parsed and which have not. Parsing failure may be because a parser has not been written for those sections, or because the syntax varies between different problems and runs. The parser writer can then determine whether the un-parsed sections are important enough to devote effort to, or whether they are of minor importance and can be effectively deleted.

The process is highly iterative. The parser templates do not cover all possible document sections and initially some parts remain unparsed. The parsers are then amended and re-run; it is relatively simple in XML to determine which parts still need work.

Currently (2011-06) there are about 200 templates for NWChem, 150 for Gaussian and a small number for Jaguar, GAMESS-UK, GAMESS(US), AMBER and MOPAC http://openmopac.net. Each time a parse fails, the section is added as a failing unit test to the template and these also act as tutorial material and a primary source of semantics for the dictionary entries.

Quixote is designed as a bottom-up community project and co-ordinated through the modern metaphors of wikis, mailing lists, Etherpads and distributed autonomous implementations. The primary entry point is currently http://quixote.wikispot.org/ which gives details of current resources and how to get involved.

### Quixote components

#### JUMBO-Converters

The JUMBO-Converters are based on a templating approach, matching the observed output to an abstraction of the QC concepts. They have been hand-crafted for a number of well-structured output files (Gaussian archive files, MOPAC and various punchfiles) but the emphasis is now on writing JUMBO-Parsers for the logfiles for each code. We have explored a wide range of technologies for parsing logfiles including machine learning, formal grammars (lex/yacc), ANTLR http://www.antlr.org/, but all of these have problems when confronted with unexpected output, variations between implementations, error messages and many other irregularities. The JUMBO-Parser will not be described in detail here but in essence consists of the following approach:

• Recognition of common document *fragments *in the logfile (*e.g*., tables of coords, eigenvalues, atomic charges, *etc*.) which appear to be produced by record-oriented (FORTRAN format) routines in the source code. We create a *template *for each such *chunk*, which contains *records*, with regexes for each record that we wish to match and from which we will extract information. These templates can be nested, often representing the internal structure of the program (*e.g*., nested subroutine calls).

• Each template is then used to match any chunks in the document, which are then regarded as completed and unavailable to other templates. The strategy allows for nesting and a small amount of back-tracking.

• Chunks of document that are not parsed may then be extracted by writing additional parsers, very often to clean up records such as error messages or timing information.

At the end of this process a good parse will results in a highly-structured document with CML module providing the structure and CML scalar, array and matrix providing the individual fields http://quixote.wikispot.org/Tutorials_and_problems.

This document is rarely fit for purpose in Quixote or other CML conventions and a second phase of transformation is applied. This carries out the following:

• Removal of unwanted fields.

• Removal of unnecessary hierarchy (often an artifact of the parsing strategy)

• Addition of dictRefs to existing dictionaries

• Addition of units (often not explicitly mentioned in the logfile but known to the parser writer)

• Grouping of sibling elements into a more tractable structure (unflattening)

• Annotation of modules to reflect semantic purpose, *e.g*., initial coordinates, optimizations, *etc*.

• Re-structuring of the modules in the parsed output to fit the *compchem *convention http://www.xml-cml.org/convention/compchem

This is carried out by a domain-specific declarative language which makes heavy use of XPath and a core set of Java routines for generic operations (delete/create/move elements, transform (matrix/molecule/strings etc.)). This approach means that failures are relatively silent (a strange document does not crash the process) and that changes can be made external to the software (by modifying the transformation files). As with the templates this should make it easier for the community to maintain the process (*e.g*. when new syntax or vocabulary occurs).

A typical template is shown in Appendix A.

JUMBO-Parser has been designed for portability, in that most of the instructions are declarative (XML). It still requires the JUMBO-Parser interpreter to be ported, but this is written in mainstream Java and should not be particularly problematic for most object-oriented targets such as Python, C++ and C#. To help in the parsing, there are a large number of unit and regression tests.

#### CML Conventions and Dictionaries

The final output is CML compliant to the *compchem *convention and validated against the current validator http://validator.xml-cml.org/. The dictionaries are in a constant state of update and consist of a reference implementation on the CML site and a working dictionary associated with the JUMBO-Converters distribution. As concepts are made firm in the latter, they are transferred to the reference dictionary.

The current compchem dictionary is shown in Appendix B. It contains about 90 terms which are independent of the codes. We expect that about the same amount again will be added to deal with other properties and solid state concepts.

#### Lensfield2

Lensfield2 https://bitbucket.org/sea36/lensfield2/ is a tool for managing file transformation workflows and can be thought of as a make for data.

Lensfield2 requires a build file, defining the various sets of input files and the conversions to be applied to them. Like make, for instance, Lensfield2 is able to detect when files have changed, and update the products of conversions depending on them. However, unlike make where this is just done through comparison of files 'last-modified times, Lensfield2 records the complete build-state, so is able to detect any change in configuration, such as when the parameterisation of builds has changed, and when versions of tools involved in the various steps of the workflow are updated or if intermediate files are altered.

Lensfield2 is designed to run workflow steps written in Java and build using Apache Maven http://maven.apache.org/, utilising Maven's dependency management system to pull in the required libraries for each build step.

Lensfield2 has been successfully used in running the parser and subsequent software over the 40,000 files in the test datasets 1-4 (*v.i*.).

#### RESTful uploading

It is important that the methods for "uploading" and "downloading" files are as flexible as possible. Some collaborators may not have privilleges to run their own server, so they need to be able to upload material to a resource run by other collaborators. However, if the protocols are complex then they may be put off taking part. Similarly, others may wish to delegate this to software agents which poll resources and aggregate material for uploading. Similar variability exists in the download process. Web-based collaborators are becoming used to very lightweight solutions such as Dropbox http://www.dropbox.com/ where files can be uploaded, and where permitted, downloaded by anyone. We do not expect a single solution to cover everything, and the more emphasis on security, the more effort required. In this phase of Quixote, we are publishing our work to the whole world and do not expect problems of corruption or misappropriation. We have therefore relied on simple proven solutions such as RESTful systems. Some of this is covered in the semantic architecture paper in this issue, and here we simply illustrate that initial systems at Cambridge have been implemented with AtomPub http://tools.ietf.org/html/rfc5023. Because the academic repository system has invested effort in the SWORD system http://www.ukoln.ac.uk/repositories/digirep/index/SWORD (which runs over AtomPub), this allows us to deposit/upload aggregations of files.

#### Chempound repository

**Figure 3:**
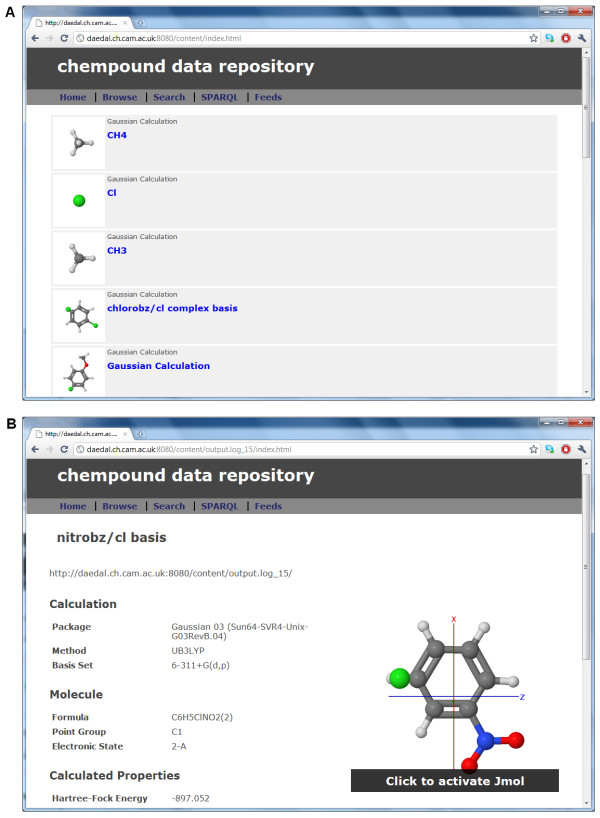
**Chempound repository graphical interface**. Chempound accepts either converted compchem CML or logfiles (which are then parsed by the JUMBO converters into compchem CML). The entries are indexed on 4 main criteria: (I) environment (program, host, dates, *etc*.) (II) initialization (molecular structure, basis sets, methods, algorithms, parameters, *etc*.) (III) calculation (the progression of optimization) (IV) finalization (molecular structure, properties, times, *etc*.) (a) Each entry is displayed with a thumbnail and key metadata (b) Properties and parameters for each entry, all searchable through SPARQL endpoint.

Quixote is built on CML compchem and, in our system, is further transformed to provide RDF used for accessing subcomponents and expressing searches. The Chempound (chem#) repository system https://bitbucket.org/chempound/[28] (see Figure 3) has been built to support this. We expect that the first wave of distributed repositories will be using Chempound, and a publically accessible prototype repository is already in use within the Quixote project http://quixote.ch.cam.ac.uk/

#### Institutional repositories, DSpace

Institutional repositories (running software such as DSpace http://www.dspace.org/ or Fedora http://fedora-commons.org/) may be responsible for storing the raw output files that are transformed into CML by the JUMBO-Converters. Alongside, they will also store basic metadata (authorship, usage rights, related works, *etc*.).

This usage of institutional repositories distributes data management responsibilities among the institutions where the creators of the raw output files work. This provides an efficient basic data management support to the creators, and lets topic-specific repositories (such as Quixote's chem#) to focus on leveraging the specialized CML semantics extracted from the raw files, while still linking back to the original raw files at the institutional repositories. This schema also favors re-use of the same primary data by different specialized research topic repositories.

Yet antother temporary advantage of this approach is that, as the data collection increases, resource discoverability becomes a real challenge - even for the researcher herself. Even if much data can be extracted from the datafiles, some title and description metadata could be very useful to issue searches and can be provided by the person submitting the files to the repository. In the development phase, other researchers - as well as the dataset creator - would be able to discover and access a given unprocessed dataset without needing to wait for it to get processed and transferred into the final Chempound data repository.

Designing a DSpace-based raw data repository will also allow for defining a *de facto *standardized metadata collection for compchem data description that may be very useful for harmonisation of data description in this specific research area - and might eventually evolve into some kind of standard for the discipline. At the present stage, we have done some preliminary work along metadata collection definition. A set of metadata has been defined and is being discussed in order to provide thorough descriptions of raw compchem datasets (potentially extendable to data from other research areas). Once the metadata set for bibliographical description of raw datasets is agreed, fields contained therein will be mapped to existing or new qualified DublinCore (QDC) metadata and a draft format will thus be defined. This format will be implemented at a DSpace-based repository, where trial-and-error storing loops with real datasets will be performed for metadata collection completion and fine -tuning - besides accounting for particular cases.

#### Avogadro

Avogadro is an open source, cross-platform desktop application to manipulate and visualize chemical data in 3D. It is available on all major operating systems, and uses Open Babel for much of its file input and output as well as basic forcefields and cheminformatics techniques. Avogadro was already capable of downloading chemical structures from the NIH structure resolver service, editing structures and optimizing those structures.

Input generation from these structures is present for many of the major computational chemistry codes Quixote targets such as GAMESS(US), GAMESS-UK, Gaussian, NWChem, MOPAC and others. These dialogs allow the user to change input parameters before producing input files to be run by the code. The output files from several of these codes can also be read directly, this functionality was recently split out into OpenQube - a library to read quantum computational code log files, and calculate molecular orbitals, electron density and other output.

Ultimately, much of this functionality will move into the Quixote parsers, with the OpenQube library concentrating on multithreaded calculation of electronic structure parameters. A native CML reader plugin has also been developed for Avogadro, to read in CML files directly and display the tree structure allowing visual exploration of CML files. As JUMBO and other tools can extract electronic structure, spectra and vibrational data, this plugin is being developed to extract them from the CML document.

Avogadro is already network aware, with a network fetch extension interacting with the NIH structure resolver and the Protein Data Bank (PDB). Experimental support for interacting with a local queue manager is also being actively developed, sending input files to the queue manager, and retrieving log files one the calculation is complete. Some data management features are being added, and as Chempound has a web API a plugin for upload, searching and downloading of structures will be added. A MongoDB-based application has been prototyped, using a document store approach to storing chemical data. This approach coupled with Chempound repositories and seamless integration in the GUI will significantly lower barriers for both deposition and retrieval of relevant computational chemistry output.

Avogadro forms a central part of the computational chemistry workflow, but is in desparate need of high quality chemical data. The data available from existing online chemical repositories is a good start, but having high quality, discoverable computational chemistry output would significantly improve efficiency in the field. Widespread access to optimized chemical structures using high level theories and large basis sets would benefit everyone from teaching right through to academic research and industry.

### Installing a Quixote repository

The Quixote system is based on the Chempound package, which provides a complete set of components for ingestion of CML, conversion to RDF and customisable display of webpages (using Freemarker templates). This installation has already been satisfactorily carried out at Zaragoza in less than 24 hours using the current Chempound distribution http://bitbucket.org/chempound and a number of calculations have been ingested. The Chempound system contains customisable modules for many types of chemical object and, in this case, is supported by the compchem module. This provides everything necessary for the default installation but, if customisation is required, the configuration and resource files in compchem-common, compchem-handler and compchem-importer can be edited. Chempound uses the SWORD2 protocol for ingest and so can accept input from any SWORD2- compliant client system.

## Results and Discussion

The Quixote project can manage input and output from any of the main compchem packages including plane-wave and solid-state approaches. The amount of semantic information in the output files can vary from a relatively small amount of metadata for indexing to a complete representation of every information output in the logfile. The community can decide at which point on the spectrum it wishes to extract information and can also retrospectively enhance this by running improved parsers and converters over the archived logfiles and output files.

The current test datasets in the Murray-Rust group are generated by parsing existing logfiles into CML using the JUMBO-Converters software. The amount of detail depends at the moment on the amount of effort that has been put into the parser. The current project is working hard to ensure inter-operability of dictionary terms and concepts by collating a top-level dictionary resource. When this is complete, the files will be re-parsed to reflect the standard semantics.

In the first pass, with the per-code parsers, we have been able to get a high conversion rate and a large number of semantic concepts from the most developed parsers. The use cases below represent work to date showing that the approach is highly tractable and can be expected to scale across all types of compchem output and types of calculation.

A typical final CML document (heavily truncated for brevity) is shown in Appendix C. This shows the structure of jobs and the typical fields to be found in most calculations.

### Test dataset 1

The first use case consisted of 1095 files in Gaussian logfile format contributed by Dr. Anna Croft of the University of Bangor. These were deliberately sent without any human description with the challenge that we could use machine methods to determine their scope and motivation. We have applied the JUMBO-Parser to these, of which all except 5 converted without problems. The average time for conversion was between 3-10 seconds depending on the size of file. These files have now been indexed, mainly from the information in the archive section of the logfile but also with the initial starting geometry and control information. A large number of the files appear to be a systematic study of the attack by halogen radicals on aromatic nuclei.

### Test dataset 2

This use case comprised of over 5000 files which Henry Rzepa and collaborators have produced over the years and which have been stored Openly in the Imperial College repository (helix). They are much more varied than the Croft sample and include studies on Möbius computational chemistry, transitional metal complexes and transition state geometries. A considerable proportion of the files emanate from student projects, many of which tackle hitherto novel chemical problems. It is our intention to create a machine-readable catalogues of these files and to determine from first principles their content and, where possible, their intent.

### Test dataset 3

The NWChem distribution (NWChem-6.0) contains a directory (/QA/tests/) with a large number (212) of varied quality assurance tests for the software. All except 18 of these have been converted satisfactorily. One problem encountered was that the parser had used a large number of regexes which, when concatenated, scaled exponentially, so that some of the conversions took over a minute. We are now re-writing the parser to use linear time methods. These files cover a wider range of chemistry than the Croft and Rzepa contributions, as many of them use plane-wave calculations on solid state problems.

### Test dataset 4

In the group of Pablo Echenique, at the Institute of Physical Chemistry "Rocasolano" (CSIC) and the University of Zaragoza, a large number of calculations were performed in peptide systems using the Gaussian quantum chemistry package. These calculations represent an exhaustive study (whose results and aims have been discussed elsewhere [14]), of more than 250 *ab initio *potential energy surfaces (PESs) of the model dipeptide HCO-L-Ala-NH_2_. The model chemistries investigated are constructed as homo- and heterolevels involving possibly different RHF and MP2 calculations for the geometry and the energy. The basis sets used belong to a sample of 39 representants from Pople's split-valence families, ranging from the small 3-21G to the large 6-311++G(2 df, 2 pd). The conformational space of this molecule is scanned by defining a regular 12×12 grid from -165° to 165° in 30° steps in the 2D space spanned by its Ramachandran angles *ϕ *and *ψ*. This totals more than 35000 Gaussian logfiles, all generated at the standard level of verbosity, some of them corresponding to single-point energy calculations, some of them to energy optimizations. The use of JUMBO-converters through Lensfield 2 has allowed to parse the totality of these files, through a complicated folder tree, generating the corresponding raw XML and structured compchem CML with a very high rate of captured concepts. The total time required to do the parsing was about five hours in an iMac desktop machine with a 2.66 GHz Intel Core 2 Duo processor, and 4 GB of RAM memory, running the Mac OS X 10.6.7 operating system.

### Quixote repository at Cambridge

The first repository (Figure 3) has been built at Cambridge http://quixote.ch.cam.ac.uk and has been viewable and searchable. In the spirit of Quixote this is not intended to be a central permanent resource but one of many repositories. It is available for an indefinite time as a demonstration of the power and flexibility of the system but not set up as a permanent "archive". It may be possible to couple such repositories to more conventional archive-oriented repositories which act as back-end storage and preservation.

## Conclusions

Each day, countless calculations are run by thousands of computational chemistry researchers around the world, on everything from ageing, dusty desktops to the most powerful supercomputers on the planet. It might be supposed that this would lead to a deluge of valuable data, but the surprising fact remains that most of this data, if it is archived at all, usually lies hidden away on hard disks or buried on tape backups; often lost to the original researcher and never seen by the wider chemistry community at all.

However, it is widely accepted that if the results of all these calculations were publicly accessible it would be extremely valuable as it would:

• avoid the costly duplication of results,

• allow different codes to be easily validated and benchmarked,

• provide the data required for the development of new methods,

• provide a valuable resource for data mining,

• provide an easy, automated way of generating and archiving supporting information for publications.

In the rare cases when data is made openly available, the output of calculations are inevitably produced in a code-specific format; there being no currently accepted output standard. This means that interpreting or reusing the data requires knowledge of the code, or the use of specific software that understands the output. A standard semantic format will:

• allow tools, (*e.g*. GUIs) to operate on the input and output of any code supporting the format, vastly increasing their utility and range,

• enable different codes to interoperate to create complex workflows,

• additionally, if a semantic model underlies the format, data can easily be validated.

The benefits of a common data standard and results databases are obvious, but several previous efforts have failed to address them, largely because of an inability to settle on a data standard or provide any useful tools that would make it worthwhile for code developers to expend the time to make their codes compatible.

The Quixote project aims to tackle both of these problems in a pragmatic way, building an infrastructure that can be used to both archive and search calculations on a local hard-drive, or expose the data on publicly accessible servers to make it available to the wider community.

The vision with which we started the Quixote project some months ago is one in which all data generated in computational QC research projects is used with maximal efficiency, is immediately made available online and aggregated into global search indexes, a vision in which no work is duplicated by researchers and everyone can get an overall picture of what has been calculated for a given system, for a given scientific question, in a matter of minutes, a vision in which all players collaborate to achieve maximum interoperability between the different stages of the scientific process of discovery, in which commonly agreed, semantically rich formats are used, and all publications expose the data as readable and reusable supplementary material, thus enforcing reproducibility of the results; a vision in which good practices are wide spread in the community, and the greatest benefit is earned from the effort invested by everyone working in the field.

With the prototype presented in this article, which has been validated by real use cases, we believe this vision is beginning to be accomplished.

The methodological approach in Quixote is novel: The data standard will be consolidated around the tools and encourage its adoption by providing code and tool developers with an obvious reason for adopting the data standard; the "If you build it, they will come" approach. The project is rooted in the belief that scientific codes and data should be "Open", and we are therefore focussing our efforts on using existing Open Source solutions and standards where possible, and then developing any additional tools within the project. The Quixote project is itself completely Open, de-centralised and community-driven. It is composed of passionate researchers from around the globe that are happy to collaborate with anyone who shares our aims.

## Competing interests

The authors declare that they have no competing interests.

## Authors' contributions

SA has participated in the design of the Quixote system, is the main developer of Chempound and collaborated in the development of the compchem dictionaries and conventions. PdeC has written the manuscript, and collaborated in the design of the D-Space-based soluion for metadata. PE has written the manuscript, participated in the design of the Quixote system and help develop some of the tools contained in it. JE has written the manuscript, participated in the design of the Quixote system and help develop some of the tools contained in it. MH has written the manuscript, participated in the design of the Quixote system and is a core developer of Avogadro. PM-R has written the manuscript, participated in the design of the Quixote system and he has been the main developer of the software tools. PS has written the manuscript, and collaborated in the design of the Quixote system. JTh has written the manuscript, participated in the design of the Quixote system and help develop some of the tools contained in it. JTo has participated in the design of the Quixote system, developed the CML validator and collaborated in the development of the compchem dictionaries and conventions. All authors have read and approved the final manuscript.

## Appendixes

### Appendix A. Template for parsing a link from Gaussian log files

A template to parse the output from the 601 link output in Gaussian logfiles. (The code for beta eigenvalues has been omitted for clarity.)

<!-- the template selects a chunk of text beginning with "pattern" and ending

      with endPattern. -->

<template id="l601.alphabetaeigen" pattern="\s*(Alpha|Beta)\s+(occ|virt)\. eigenvalues.*"

   repeat="*"

   endPattern="\s[^A][^B].*">

   <!-- an example of input which also acts as a unit test -->

   <comment class="example.input" id="l601.alphabeta">

Alpha occ. eigenvalues -- -10.17514 -0.68437 -0.38581 -0.38581 -0.38581

Alpha virt. eigenvalues -- 0.11292 0.17036 0.17036 0.17036 0.53917

Alpha virt. eigenvalues -- 0.53917 0.53917 0.88316 0.88316 0.88316

Alpha virt. eigenvalues -- 0.91927 1.09380 1.66027 1.66027 2.21731

Alpha virt. eigenvalues -- 2.21731 2.21731 4.16488

   </comment>

   <!-- all lines are parsed with a single recordReader. The trailing part of the line is

         captured into an array and names with the leading string -->

   <record id="eigen" repeat="*">\s*{X, g:name}\s*eigenvalues \-\-{1_5F, g:eigen}</record>

   <!-- rename the dictRef to conform to dictionary and to CML syntax -->

   <transform process="addDictRef" xpath=".//cml:array[@cmlx:temp=' Alpha occ.']" value="cc:alphaocc"/>

   <transform process="addDictRef" xpath=".//cml:array[@cmlx:temp='Alpha virt.']" value="cc:alphavirt"/>

   <!-- stitch lines together -->

   <transform process="joinArrays" xpath=".//cml:array[@dictRef='g:alphaocc']"/>

   <transform process="joinArrays" xpath=".//cml:array[@dictRef='g:alphavirt']"/>

   <!-- expected output (can also be used for unit testing). Note that the result is

         labelled with the chunk number (l601) as well as the -->

   <comment class="example.output" id="l601.alphabeta">

      <module cmlx:templateRef="l601.alphabetaeigen" xmlns="http://www.xml-cml.org/schema" xmlns:cmlx="http://www.xml-cml.org/schema/cmlx">

         <array dataType="xsd:double" size="5" dictRef="cc:alphaocc">-10.17514 -0.68437 -0.38581 -0.38581 -0.38581</array>

         <array dataType="xsd:double" size="18" dictRef=cc:alphavirt">

            0.11292 0.17036 0.17036 0.17036 0.53917 0.53917 0.53917 0.88316 0.88316

            0.88316 0.91927 1.0938 1.66027 1.66027 2.21731 2.21731 2.21731 4.16488</array>

      </module>

   </comment>

### Appendix B. Dictionary for Computational chemistry

The current dictionary for (code-independent) computational chemistry. A few entries are shown in full; most show the id's and the terms. The full dictionary is maintained within the current Bitbucket content.

<!-- the dictionary element contains the namespaces, convention,

      prefix and title required for the dictionary -->

<dictionary

   xmlns="http://www.xml-cml.org/schema" <!-- CML -->

   xmlns:cmlx="http://www.xml-cml.org/schema/cmlx" <!-- CML extensions -->

   xmlns:h="http://www.w3.org/1999/xhtml" <!-- XHTML -->

   xmlns:conventions="http://www.xml-cml.org/convention/" <!-- convention namespace -->

   xmlns:unitType="http://www.xml-cml.org/unit/unitType/" <!-- CML unitType namespace -->

   xmlns:si="http://www.xml-cml.org/unit/si/" <!-- SI units -->

   xmlns:nonSi="http://www.xml-cml.org/unit/nonSi/" <!-- other units -->

   xmlns:dc="http://purl.org/dc/elements/1.1/"> <!-- Dublin Core -->

   namespace="http://www.xml-cml.org/dictionary/compchemhttp:///" <!-- namespace of the dictionary -->

   convention="conventions:dictionary" <!-- convention for the dictionary -->

   dictionaryPrefix="compchem" <!-- default prefix for compchem -->

   title="Computational chemistry concepts - CompChem concepts"

<description>

   <h:p>Top level dictionary for computational chemistry</h:p>

   <h:p>

      Concepts in this dictionary are general throughout computational

      chemistry and are used extensively in the

      <h:a href="http://www.xml-cml.org/convention/compchem/">compchem convention</h:a>

      to describe the structure of

      computational chemistry.

   </h:p>

   <h:p>NOTE: Many of the entries are still being developed. Some of

      the terms are taken from the annotations on the logfile, but some are guessed.

      The dictionary is intended for public comment

      and development, not absolute approval. Units and unitTypes are often unknown or very difficult

      and may take many discussions to agree. (Remember the crystallographers

      did not build CIF in a day!)</h:p>

</description>

<dc:contributor>Weerapong Phadungsukanan</dc:contributor>

<dc:contributor>Peter Murray-Rust</dc:contributor>

<dc:contributor>Joe Townsend</dc:contributor>

<!-- all the current dictionary entries are listed, but most are

      truncated in this display for brevity -->

<!-- document structure entries -->

<entry id="jobList" term="job list" unitType="unitType:none">

   <definition>

      <h:p>A list of computational jobs</h:p>

   </definition>

   <description>

      <h:p>

         A quantum chemistry calculation is often comprised of a series

         of subtasks,

         <h:i>e.g.</h:i>

         coarse optimisation -> fine optimisation -> NMR Spectrum

         Analysis; this is

         because most quantum chemistry software packages are designed to be

         modularised and only to perform a single task at a time. The

         joblist concept

         is introduced to capture these series of successive subtasks and links

         the information from one subtask to the next subtask.

      </h:p>

   </description>

</entry>

<entry id="job" term="job" unitType="unitType:none">

   <definition>

      <h:p>A job or computational task</h:p>

   </definition>

<description>

      <h:p>

         The job concept represents a computational job performed by quantum

         chemistry

         software, e.g. geometry optimisation job, frequency analysis job. The job

         concept is the smallest unit which can fully describe a general

         picture of

         computational modelling.

   </h:p>

   </description>

</entry>

<entry id="initialization" term="initialization" unitType="unitType:none">

   <definition>

      <h:p>A initialisation module for a computational job</h:p>

   </definition>

   <description>

      <h:p>

         An initialisation module represents the concept of the model

         parameters and

         inputs for computational job.

      </h:p>

   </description>

</entry>

<entry id="calculation" term="calculation" unitType="unitType:none">

   <definition>

      <h:p>A calculation module for a computational job</h:p>

   </definition>

   <description>

      <h:p>

         A calculation module represents the concept of the model calculation or

         optimisation or iteration processes for computational job.

      </h:p>

   </description>

</entry>

<entry id="finalization" term="finalization" unitType="unitType:none">

   <definition>

      <h:p>A finalization module for a computational job</h:p>

   </definition>

   <description>

      <h:p>

         A finalisation module represents the concept of the model results for

         computational job.

      </h:p>

   </description>

</entry>

<entry id="environment" term="environment" unitType="unitType:none">

   <definition>

      <h:p>Module holding concepts relating to environment that the job

         used or required</h:p>

   </definition>

   <description>

      <h:p>

         The computing environment concept refers to a hardware platform,

         software application,

         the operating system and any hardware and software configurations used

         in order to run

         the job or computational task. The environment also includes the

         metadata such as

         machine id, username, starting and finishing date time, tools, compilers,

         IP, etc.

      </h:p>

      <h:p>

         This information is not related to input and output of the model but is

         supplementary to

         the software application to run properly and may vary from machine to

         machine.

         Therefore, the computing environment is OPTIONAL element in the CompChem

         convention.

      </h:p>

   </description>

</entry>

<!-- entries describing data in the document -->

<!-- general entries -->

<entry id="alphae" term="Number of alpha electrons" dataType="xsd:integer"

         units="si:none" unitType="unitType:none">

   <definition>

      <h:p>Number of alpha electrons</h:p>

   </definition>

   <description>

      <h:p>In closed shell calculations this equals the number of beta

         electrons</h:p>

      <h:p class="example">5</h:p>

      <h:p class="gaussian.example">

         <h:a href="gaussian/log/templates/l301..xml">Gaussian</h:a>

         5 alpha electrons

      </h:p>

   </description>

</entry>

<entry id="betae" term="Number of beta electrons" dataType="xsd:integer" ... </entry>

<entry id="alphaocc" term="Energy of orbitals occupied by alpha electrons" ... </entry>

<entry id="betaocc" term="Energy of orbitals occupied by beta electrons" ... </entry>

<entry id="alphavirt" term="Energy of virtual orbitals for alpha electrons" ... </entry>

<entry id="betavirt" term="Energy of virtual orbitals for beta electrons"

<entry id="basis" term="Basis set" dataType="xsd:string"

         cmlx:format="" units="si:none" unitType="unitType:none">

   <definition>

      <h:p>Basis set</h:p>

   </definition>

   <description>

      <h:p class="example">6-31G(d)</h:p>

      <h:p class="gaussian.example">

         <h:a href="gaussian/log/templates/l301..xml">Gaussian</h:a>Standard basis: 6-31G(d)

      </h:p>

   </description>

</entry>

<entry id="basiscount" term="Number of basis set components...</entry>

<entry id="date" term="Date job was run" dataType="xsd:date"

         units="si:none" unitType="unitType:none">

   <definition>

      <h:p>Date job was run</h:p>

   </definition>

   <description>

      <h:p>Probably the date-time when the program started executing

         rather than when

         it was submitted to the queue.</h:p>

      <h:p class="example">2006-11-20T00:00:00Z</h:p>

   </description>

</entry>

<entry id="degfreedom" term="Degrees of freedom" dataType="xsd:integer...</entry>

<entry id="diffuse" term="Diffuse orbitals" dataType="xsd:string" ...</entry>

<entry id="dipole.magnitude" term="Electric Dipole moment magnitude"

         dataType="xsd:double" cmlx:format="" unitType="unitType:electric_dipole_moment">

   <definition>

      <h:p>Electric Dipole moment magnitude</h:p>

   </definition>

   <description>

      <h:p class="example">0.00</h:p>

      <h:p class="gaussian.example">

         <h:a href="gaussian/log/templates/l601.multipole..xml">Gaussian</h:a>

         Dipole moment (field-independent basis, Debye): × = 0.0000

         Y = 0.0000 Z = 0.0000 Tot = 0.0000

      </h:p>

   </description>

</entry>

<entry id="dipole.vector" term="Electric Dipole moment vector" ...</entry>

<entry id="dipolederiv" term="Derivatives of electric dipole"

         dataType="xsd:double" cmlx:format="cml:array">

   <definition>

      <h:p>Derivatives of electric dipole moment wrt coordinates</h:p>

   </definition>

   <description>

      <h:p>A symmetric matrix (3*N * 3*N). May be represented as a lower

         triangle with diagonal terms.</h:p>

      <h:p class="example">7.872E-4 0.0 0.0 0.0 7.871E-4 0.0 0.0 0.0 7.871E-4

         0.0733391 0.0 0.0 0.0 0.0733391 0.0 0.0 0.0 -0.1472685

         -0.1227565 0.0 0.0693303 0.0 0.0733391 0.0 0.0693303 0.0

         0.0488271 0.0243152 -0.0849119 -0.0346651 -0.0849119 -0.0737327

         -0.0600418 -0.0346651 -0.0600418 0.0488271 0.0243151 0.0849119

         -0.0346652 0.0849119 -0.0737326 0.0600418 -0.0346652 0.0600418

         0.0488271</h:p>

      <h:p class="gaussian.example">

         <h:a href="gaussian/log/templates/foo..xml">Gaussian</h:a>

      </h:p>

   </description>

</entry>

<entry id="displacement" term="Atomic vibrational displacment...</entry>

<entry id="electronicstate" term="Electronic state" dataType="xsd:string...</entry>

<entry id="forceConstants" term="Cartesian Force constants"

         dataType="xsd:double" cmlx:format="cml:array">

   <definition>

      <h:p>Cartesian Force constants</h:p>

   </definition>

   <description>

      <h:p>A symmetric matrix (3*N * 3*N) often reported as a lower

         triangle</h:p>

      <h:p class="example">0.55936081 0.0 0.55936081 0.0 0.0 0.55936081

         -0.0482856 0.0 -3.0E-8 0.04683066 0.0 -0.0482856 -5.0E-8 0.0

         0.04683066 -3.0E-8 -5.0E-8 -0.32294941 3.0E-8 5.0E-8 0.34888521

         -0.29243119 -3.0E-8 0.08631853 0.00266595 0.0 -6.7139E-4

         ...

         -0.08220885 0.0803923</h:p>

   </description>

</entry>

<entry id="forces" term="Residual forces on atoms" dataType="xsd:double...</entry>

<entry id="frameworkgroup" term="Framework group" dataType="xsd:string...</entry>

<entry id="frequency" term="Vibrational frequencies" dataType="xsd:double"

         cmlx:format="cml:array">

   <definition>

      <h:p>Vibrational frequencies</h:p>

   </definition>

   <description>

      <h:p class="example">1373.4987 1373.4987 1373.4987 1593.9619 1593.9619

         3053.5976 3163.9228 3163.9228 3163.9228</h:p>

   </description>

</entry>

<entry id="hexadecapole" term="Hexadecapole electric moment...</entry>

<entry id="hfenergy" term="energy" dataType="xsd:double...</entry>

<entry id="hostname" term="Hostname" dataType="xsd:string...</entry>

<entry id="irintensity" term="Infrared intensities" dataType="xsd:double"

         cmlx:format="cml:array">

   <definition>

      <h:p>Predicted Infrared intensities</h:p>

   </definition>

   <description>

      <h:p class="example">15.4253 15.4253 15.4253 0.0 0.0 0.0 26.421 26.421

         26.421</h:p>

   </description>

</entry>

<entry id="irrep" term="Irreducible representations of vibrations"

         dataType="xsd:string" cmlx:format="cml:array" units="si:none"

         unitType="unitType:none">

   <definition>

      <h:p>Irreducible representations of vibrations</h:p>

   </definition>

   <description>

      <h:p class="example">|T2|T2|T2|E|E|A1|T2|T2|T2|</h:p>

   </description>

</entry>

<entry id="jobdatetime.end" term="Date time for finish of job"

         dataType="xsd:date" units="si:none" unitType="unitType:none"

         cmlx:format="">

   <definition>

      <h:p>Date time for finish of job</h:p>

   </definition>

   <description>

      <h:p class="example">2006-11-20T14:40:23Z</h:p>

   </description>

</entry>

<entry id="jobname" term="job name" dataType="xsd:string...</entry>

<entry id="jobtime" term="elapsed time" dataType="xsd:date...</entry>

<entry id="keyword" term="keyword" dataType="xsd:string...</entry>

<entry id="polarizability" term="polarizability" dataType="xsd:double...</entry>

<entry id="method" term="method or functional" dataType="xsd:string...</entry>

<entry id="moi" term="moment of inertia" dataType="xsd:double"

         cmlx:format="cml:vector3">

   <definition>

      <h:p>moment of inertia</h:p>

   </definition>

   <description>

      <h:p class="example">11.47105 11.47105 11.47105</h:p>

   </description>

</entry>

<entry id="moi.eigenvectors" term="moment of inertia eigenvectors...</entry>

<entry id="molmass" term="molecular mass" dataType="xsd:double...</entry>

<entry id="nactiveatoms" term="number of active atoms" dataType="xsd:integer...</entry>

<entry id="natoms" term="number of atoms" dataType="xsd:integer...</entry>

<entry id="nucrepener" term="nuclear repulsion energy" dataType="xsd:double">

<entry id="octapole" term="octapole electric moment" dataType="xsd:double...</entry>

<entry id="pointgroup" term="pointgroup" dataType="xsd:string...</entry>

<entry id="press" term="pressure" dataType="xsd:double...</entry>

<entry id="program" term="program" dataType="xsd:string" units="si:none...</entry>

<entry id="program.date" term="date of program creation...</entry>

<entry id="quadrupole" term="quadrupole" dataType="xsd:double...</entry>

<entry id="redmass" term="reduced mass" dataType="xsd:double...</entry>

<entry id="rmsd" term="RMS deviation" dataType="xsd:double...</entry>

<entry id="rmsf" term="RMS force" dataType="xsd:double">

<entry id="rotconst" term="rotational constants" dataType="xsd:double">

<entry id="rottemp" term="rotational temperature" dataType="xsd:double...</entry>

<entry id="symmnumber" term="symmetry number" dataType="xsd:integer...</entry>

<entry id="temp" term="temperature" dataType="xsd:double">

<entry id="title" term="title" dataType="xsd:string" units="si:none...</entry>

<entry id="top" term="type of top" dataType="xsd:string" units="si:none...</entry>

<entry id="uniqatoms" term="number of unique atoms" dataType="xsd:integer...</entry>

<entry id="version" term="version of program" dataType="xsd:string...</entry>

<entry id="vibtemp" term="vibrational temperature" dataType="xsd:double...</entry>

<entry id="virtualorbs" term="virtual orbitals" dataType="xsd:string...</entry>

<entry id="zpe" term="zero-point energy" dataType="xsd:double">

<entry id="zpe.correction" term="zero-point energy correction...</entry>

<entry id="zpe.sumelectthermal" term="Thermal correction to Energy...</entry>

<entry id="zpe.sumelectthermalenthalpy" term="Thermal correction to Enthalpy...</entry>

<entry id="zpe.sumelectthermal" term="Thermal correction to Gibbs Free Energy...</entry>

<entry id="zpe.sumelectzpe" term="Sum of electronic and zero-point Energies...</entry>

<entry id="zpe.thermalcorrener" term="thermal correction energy...</entry>

<entry id="zpe.thermalcorrenthalpy" term="thermal correction to enthalpy...</entry>

<entry id="zpe.thermalcorrgfe" term="thermal correction gibbs free energy...</entry>

<entry id="formalCharge" term="Formal charge" dataType="xsd:integer">

<entry id="formula" term="formula" dataType="xsd:string...</entry>

<entry id="multiplicity" term="Spin multiplicity" dataType="xsd:integer"

         cmlx:format="" units="si:none" unitType="unitType:none">

   <definition>

      <h:p>Spin multiplicity</h:p>

   </definition>

   <description>

      <h:p class="example">1</h:p>

   </description>

</entry>

</dictionary>

### Appendix C. CML produced from a Gaussian log file

A complete semantic parse for a Gaussian log file (Dr Anna Croft, for methane CH4). The log files describes two chained jobs, the first an optimization and the second the calculation of frequencies and thermochemistry. All significant information is captured, but much is repetitious and much is omitted here for brevity. Some fields have been truncated for clarity - no precision is lost in parsing.

The complete parse can be found at

<?xml version="1.0" encoding="UTF-8"?>

<module convention="convention:compchem" xmlns="http://www.xml-cml.org/schema"

   xmlns:cmlx="http://www.xml-cml.org/schema/cmlx"

   xmlns:convention="http://www.xml-cml.org/convention/"

   xmlns:cc="http://www.xml-cml.org/dictionary/compchemhttp:///"

   xmlns:compchem="http://www.xml-cml.org/dictionary/compchemhttp:///"

   xmlns:g="http://www.xml-cml.org/dictionary/gaussian/" xmlns:xsd="http://www.w3.org/2001/XMLSchema"

   xmlns:nonsi="http://www.xml-cml.org/unit/nonSi/"

   xmlns:cml="http://www.xml-cml.org/dictionary/cml/">

   <!-- all dictRef values prefixed by cc are linked to the general compchem dictionary. The "g"

         prefix links to a code-specific dictionary -->

   <!-- the log file describes two jobs under a parent jobList -->

      <module id="jobList1" dictRef="cc:jobList">

      <!-- the first job --

      <module dictRef="cc:job" id="job1">

         <!-- mandatory environment module -->

         <module id="environment" dictRef="cc:environment">

               <parameterList>

                  <parameter dictRef="cc:program"><scalar >Gaussian 03</scalar></parameter>

                  <parameter dictRef="cc:hostname"><scalar >GINC-DEEPTHOUGHT</scalar></parameter>

                  <parameter dictRef="cc:jobname"><scalar >WWW-DATA</scalar></parameter>

                  <parameter dictRef="cc:date">

                     <scalar dataType="xsd:date">2006-11-20T00:00:00Z</scalar></parameter>

                  <parameter dictRef="cc:title"><scalar >CH4</scalar></parameter>

                  <parameter dictRef="cc:version"><scalar >x86-Linux-G03RevB.04</scalar></parameter>

                  <parameter dictRef="cc:run.date"><scalar >20-Nov-2006</scalar></parameter>

                  <parameter dictRef="cc:program"><scalar >Gaussian 03</scalar></parameter>

                  <parameter dictRef="cc:program.date"><scalar >2-Jun-2003</scalar></parameter>

                  <parameter dictRef="cc:version"><scalar >x86-Linux-G03RevB.04</scalar></parameter>

               </parameterList>

</module>

   <!-- mandatory intialization module -->

   <module id="initialization" dictRef="cc:initialization">

      <parameterList>

         <parameter dictRef="cc:nactiveatoms"><scalar dataType="xsd:integer">5</scalar></parameter>

         <parameter dictRef="cc:natoms"><scalar dataType="xsd:integer">5</scalar></parameter>

         <parameter dictRef="cc:betae"><scalar dataType="xsd:integer">5</scalar></parameter>

         <parameter dictRef="cc:alphae"><scalar dataType="xsd:integer">5</scalar></parameter>

         <parameter dictRef="cc:basiscount"><scalar dataType="xsd:integer">23</scalar></parameter>

         <parameter dictRef="cc:diffuse"><scalar >(6D, 7F)</scalar></parameter>

         <parameter dictRef="cc:basis"><scalar >6-31G(d)</scalar></parameter>

         <parameter dictRef="cc:degfreedom"><scalar dataType="xsd:integer" >1</scalar></parameter>

         <parameter dictRef="cc:frameworkgroup"><scalar >TD[O(C),4C3(H)]</scalar></parameter>

         <parameter dictRef="cc:pointgroup"><scalar >TD</scalar></parameter>

         <parameter dictRef="cc:method"><scalar >RB3LYP</scalar></parameter>

         <parameter dictRef="cc:basis"><scalar >6-31G(d)</scalar></parameter>

         <!-- Gaussian specific keyword -->

         <parameter dictRef="g:operation"><scalar >FOpt</scalar></parameter>

         <parameter dictRef="g:keyword"><scalar >#N</scalar></parameter>

         <parameter dictRef="g:keyword"><scalar >B3LYP/6-31G(D)</scalar></parameter>

         <parameter dictRef="g:keyword"><scalar >OPT</scalar></parameter>

         <parameter dictRef="g:keyword"><scalar >FREQ</scalar></parameter>

      </parameterList>

   <!-- mandatory input molecule -->

   <molecule id="mol.l202.orient" >

      <atomArray>

         <atom id="a1" elementType="C" x3="0.0" y3="0.0" z3="0.0"/>

         <atom id="a2" elementType="H" x3="0.0" y3="0.0" z3="1.113"/>

         <atom id="a3" elementType="H" x3="1.049347" y3="0.0" z3="-0.371"/>

         <atom id="a4" elementType="H" x3="-0.524673" y3="-0.908761" z3="-0.371"/>

         <atom id="a5" elementType="H" x3="-0.524673" y3="0.908761" z3="-0.371"/>

      </atomArray>

      <formula formalCharge="0" concise="C 1 H 4"/>

      <bondArray>

         <bond atomRefs2="a1 a2" id="a1_a2" order="S"/>

         <bond atomRefs2="a1 a3" id="a1_a3" order="S"/>

         <bond atomRefs2="a1 a4" id="a1_a4" order="S"/>

         <bond atomRefs2="a1 a5" id="a1_a5" order="S"/>

      </bondArray>

   </molecule>

   <module id="otherComponents" dictRef="cc:userDefinedModule"> ...</module>

   </module>

   <!-- mandatory calculation module -->

   <module id="calculation" dictRef="cc:calculation">

      <module id="otherComponents" dictRef="cc:userDefinedModule">

         <scalar dataType="xsd:double" dictRef="cc:nucrepener">13.1577484238</scalar>

         <module >

            <scalar dictRef="g:stoichiometry">CH4</scalar>

            <scalar dictRef="cc:frameworkgroup">TD[O(C),4C3(H)]</scalar>

            <scalar dataType="xsd:integer" dictRef="cc:degfreedom">1</scalar>

         </module>

      <module >

         <array dataType="xsd:double" dictRef="cc:rotconst" size="3">

            157.5433763 157.5433763 157.5433763</array>

      </module>

      <scalar dataType="xsd:double" dictRef="cc:nucrepener" >13.4043316016</scalar>

      <!-- module/link specific information -->

      <module >

      <list >

         <array dataType="xsd:integer" dictRef="cc:adapted" size="4">8 5 5 5</array>

         <array dictRef="cc:symm" size="4">A B1 B2 B3</array>

      </list>

         <scalar dataType="xsd:integer" dictRef="cc:basiscount">23</scalar>

         <scalar dataType="xsd:integer" dictRef="g:primbasis">44</scalar>

         <scalar dataType="xsd:integer" dictRef="cc:cartesianbasis">23</scalar>

         <scalar dataType="xsd:integer" dictRef="cc:alphae">5</scalar>

         <scalar dataType="xsd:integer" dictRef="cc:betae">5</scalar>

         <scalar dataType="xsd:integer" dictRef="cc:natoms">5</scalar>

         <scalar dataType="xsd:integer" dictRef="cc:nactiveatoms">5</scalar>

         <scalar dataType="xsd:integer" dictRef="cc:uniqatoms">2</scalar>

         <scalar dataType="xsd:double" dictRef="g:sfac">5.66</scalar>

         <scalar dataType="xsd:integer" dictRef="g:natfmm">60</scalar>

         <scalar dictRef="g:big">F</scalar>

   </module>

...

<!-- convergence information -->

<module dictRef="cc:userDefinedModule">

   <list >

      <array dictRef="g:item"/>

      <array dataType="xsd:double" dictRef="g:val" size="4">4.5E-4 3.E-4 .0018 .0012</array>

      <array dataType="xsd:double" dictRef="g:threshold" size="4">0.0 0.0 0.0 0.0</array>

      <array dictRef="g:converged" size="4">NO NO YES YES</array>

      </list>

  </module>

  </module>

</module>

<!-- mandatory finalization module -->

<module id="finalization" dictRef="cc:finalization">

   <propertyList>

      <property dictRef="cc:jobtime"><scalar >PT16.200S</scalar></property>

      <property dictRef="cc:jobdatetime.end">

         <scalar dataType="xsd:date">2006-11-20T14:40:23Z</scalar></property>

      <property dictRef="cc:electronicstate"><scalar >1-A1</scalar></property>

      <property dictRef="cc:hfenergy">

         <scalar dataType="xsd:double" units="nonsi:hartree">-40.5183892</scalar></property>

      <property dictRef="cc:rmsd">

         <scalar dataType="xsd:double" units="nonsi:unknown">2.782E-9</scalar></property>

      <property dictRef="cc:rmsf">

         <scalar dataType="xsd:double" units="nonsi:unknown">8.238E-8</scalar></property>

      <property dictRef="cc:dipole">

<array dataType="xsd:double" units="nonsi:debye" size="3">0.0 0.0 0.0</array></property>

      <property dictRef="cc:multipole">

<list >

<array dataType="xsd:double" dictRef="cc:dipole" size="3">0.0 0.0 0.0</array>

         <scalar dataType="xsd:double" dictRef="x:dipole">0.0</scalar>

<array dataType="xsd:double" dictRef="cc:quadrupole" size="6">

            -8.3036 -8.3036 -8.3036 0.0 0.0 0.0</array>

<array dataType="xsd:double" dictRef="cc:octapole" size="10">0.0 ... -0.7195</array>

<array dataType="xsd:double" dictRef="cc:hexadecapole" size="15">-16.22 ... .0</array>

</list></property>

      <property dictRef="cc:virtualorbs"><array delimiter="|" size="18">

   |(A1)|(T2)|(T2)|...|(T2)|(A1)|</array></property>

</propertyList>

<!-- final molecule -->

<molecule id="mol9999">

   <atomArray>

      <atom id="a1" elementType="C" x3="0.0" y3="0.0" z3="0.0"/>

      <atom id="a2" elementType="H" x3="9.95E-8" y3="1.925E-7" z3="1.09326594"/>

      <atom id="a3" elementType="H" x3="1.0307409799" y3="1.096E-7" z3="-0.3644220738"/>

      <atom id="a4" elementType="H" x3="-0.5153703892" y3="-0.8926480531" z3="-0.3644217759"/>

      <atom id="a5" elementType="H" x3="-0.5153706902" y3="0.892647751" z3="-0.3644220902"/>

   </atomArray>

   <formula formalCharge="0" concise="C 1 H 4" dictRef="cc:formula.calc"/>

   <bondArray>

      <bond atomRefs2="a1 a2" id="a1_a2" order="S"/>

      <bond atomRefs2="a1 a3" id="a1_a3" order="S"/>

      <bond atomRefs2="a1 a4" id="a1_a4" order="S"/>

      <bond atomRefs2="a1 a5" id="a1_a5" order="S"/>

   </bondArray>

   <formula concise="C 1 H 4" dictRef="cc:formula.user"/>

  </molecule>

 </module>

</module>

<!-- final job (takes input from job 1) -->

<module dictRef="cc:job" id="job2">

   <module id="environment" dictRef="cc:environment">

      <parameterList>

         <parameter dictRef="cc:date">

            <scalar dataType="xsd:date">2006-11-20T00:00:00Z</scalar></parameter>

         </parameterList>

      </module>

      <module id="initialization" dictRef="cc:initialization">

         <parameterList>

            <parameter dictRef="g:keyword"><scalar >GEOM=ALLCHECK</scalar></parameter>

            <parameter dictRef="g:keyword"><scalar >GUESS=READ</scalar></parameter>

            <parameter dictRef="g:keyword"><scalar >SCRF=CHECK</scalar></parameter>

            <parameter dictRef="g:keyword"><scalar >GENCHK</scalar></parameter>

            <parameter dictRef="g:keyword"><scalar >RB3LYP/6-31G(D)</scalar></parameter>

            <parameter dictRef="g:keyword"><scalar >FREQ</scalar></parameter>

         </parameterList>

      </module>

      <module id="calculation" dictRef="cc:calculation">

         <module id="otherComponents" dictRef="cc:userDefinedModule">

         <scalar dataType="xsd:double" dictRef="cc:nucrepener" >13.3952533229</scalar>

         </module>

      </module>

<!-- final coordinates and properties -->

<module id="finalization" dictRef="cc:finalization">

   <propertyList>

      <property dictRef="cc:jobtime"><scalar >PT12.700S</scalar></property>

      <property dictRef="cc:jobdatetime.end">

         <scalar dataType="xsd:date">2006-11-20T14:40:36Z</scalar></property>

      <property dictRef="g:l601.pol.exact">

      <array dataType="xsd:double" size="6">12.353 .0 12.353 .0 .0 12.353</array></property>

      <property dictRef="cc:frequencies">

         <table id="l716.forcematrix">

            <array dataType="xsd:integer" dictRef="x:serial" size="9">1 2 3 4 5 6 7 8 9</array>

            <array delimiter="|" dictRef="cc:irrep" size="9">|T2|T2|T2|E|E|A1|T2|T2|T2|</array>

            <array dataType="xsd:double" dictRef="cc:frequency" size="9">

               1373.49 1373.49 1373.49 1593.96 1593.96 3053.59 3163.92 3163.92 3163.92</array>

            <array dataType="xsd:double" dictRef="cc:redmass" size="9">

               1.1787 1.1787 1.1787 1.0078 1.0078 1.0078 1.1019 1.1019 1.1019</array>

            <array dataType="xsd:double" dictRef="cc:forceconst" size="9">

               1.3101 1.3101 1.3101 1.5087 1.5087 5.5368 6.4991 6.4991 6.4991</array>

            <array dataType="xsd:double" dictRef="cc:irintensity" size="9">

               15.4253 15.4253 15.4253 0.0 0.0 0.0 26.421 26.421 26.421</array>

         </table></property>

      <property dictRef="cc:thermochemistry">

         <list id="l716.thermochemistry">

         <scalar dataType="xsd:double" dictRef="cc:temp">298.15</scalar>

         <scalar dataType="xsd:double" dictRef="cc:press">1.0</scalar>

         <scalar dataType="xsd:double" dictRef="cc:molmass">16.0313</scalar>

         <matrix rows="3" columns="3" dataType="xsd:double" dictRef="cc:moi.eigenvectors">

               0.0 0.0 1.0 0.0 1.0 0.0 1.0 0.0 0.0</matrix>

            <array dataType="xsd:double" dictRef="cc:moi" size="3">11.471 11.471 11.471</array>

         <scalar dictRef="g:top">spherical</scalar>

         <scalar dataType="xsd:integer" dictRef="cc:symmnumber">12</scalar>

            <array dataType="xsd:double" dictRef="cc:rottemp" size="3">7.550 7.550 7.550</array>

            <array dataType="xsd:double" dictRef="cc:rotconst" size="3">

               157.33005 157.33005 157.33005</array>

         <scalar dataType="xsd:double" dictRef="cc:zpe" units="u:jmol-1">118752.0</scalar>

            <array dataType="xsd:double" dictRef="cc:vibtemp" size="9">

               1976.16 1976.16 1976.16 2293.35 2293.35 4393.44 4552.17 4552.17 4552.17</array>

      </list></property>

      <property dictRef="cc:zeropoint">

      <list id="l716.zeropoint">

         <scalar dictRef="cc:zpe.correction">0.04523</scalar>

         <scalar dictRef="cc:zpe.thermalcorrener">0.048094</scalar>

         <scalar dictRef="cc:zpe.thermalcorrenthalpy">0.049039</scalar>

         <scalar dictRef="cc:zpe.thermalcorrgfe">0.027907</scalar>

         <scalar dictRef="cc:zpe.sumelectzpe">-40.473159</scalar>

         <scalar dictRef="cc:zpe.sumelectthermal">-40.470295</scalar>

         <scalar dictRef="cc:zpe.sumelectthermal">-40.469351</scalar>

         <scalar dictRef="cc:zpe.sumelectthermalfe">-40.490482</scalar>

      </list></property>

      <property dictRef="cc:electronicstate"><scalar >1-A1</scalar></property>

      <property dictRef="cc:hfenergy">

         <scalar dataType="xsd:double" units="nonsi:hartree">-40.5183892</scalar></property>

      <property dictRef="cc:rmsd">

         <scalar dataType="xsd:double" units="nonsi:unknown">8.723E-11</scalar></property>

      <property dictRef="cc:rmsf">

         <scalar dataType="xsd:double" units="nonsi:unknown">8.224E-8</scalar></property>

      <property dictRef="cc:dipole">

            <array dataType="xsd:double" units="nonsi:debye" size="3">0.0 0.0 0.0</array></property>

      <property dictRef="cc:multipole">...</property>

      <property dictRef="cc:virtualorbs"><array delimiter="|" size="18">

         |(A1)|(T2)|(T2)|(T2)...|(T2)|(T2)|(A1)|</array></property>

</propertyList>

<module id="otherComponents" dictRef="cc:userDefinedModule">

   <module dictRef="cc:userDefinedModule">

            <array dataType="xsd:double" dictRef="cc:dipolederiv" units="nonsi:unknown" size="45">

               7.872E-4 0.0 0.0 0.0 7.871E-4 0.0 0.0 0.0 7.871E-4 ... 0.0488271</array>

            <array dataType="xsd:double" dictRef="cc:polarizability" units="nonsi:unknown" size="6">

               12.3528403 0.0 12.3528403 0.0 0.0 12.3528403</array>

            <scalar dictRef="cc:pointgroup">TD [O(C1),4C3(H1)]</scalar>

            <array dataType="xsd:double" dictRef="cc:forceConstants" size="120">

               0.55936081 0.0 0.55936081 0.0 0.0 0.55936081...</array>

            <array dataType="xsd:double" dictRef="cc:forces" size="15">0.0 0.0.... -5.0E-8</array>

   </module>

</module>

<!-- final molecule. Atoms may have child properties -->

<molecule id="mo19999">

   <atomArray>

   <atom id="a1" elementType="C" x3="0.0" y3="0.0" z3="0.0">

      <property dictRef="cc:vibdisplacements">

         <list>

            <array dataType="xsd:double" size="3" dictRef="cc:displacement">.0 .0 0.12</array>

            ... 9 in total ...

         </list></property>

      <property dictRef="cc:force">

         <array dataType="xsd:double" size="3">0.0 0.0 0.0</array></property>

      <property dictRef="cc:mulliken">

         <scalar dataType="xsd:double">-0.628247</scalar></property>

   </atom>

   <atom id="a2" elementType="H" x3="9.95E-8" y3="1.925E-7" z3="1.09326594">...</atom>

   <atom id="a3" elementType="H" x3="1.0307409799" y3="1.096E-7" z3="-0.3644220738">...</atom>

   <atom id="a4" elementType="H" x3="-0.5153703892" y3="-0.8926" z3="-0.3644">...</atom>

   <atom id="a5" elementType="H" x3="-0.5153706902" y3="0.8926" z3="-0.3644">...</atom>

</atomArray>

<formula formalCharge="0" concise="C 1 H 4" dictRef="cc:formula.calc"/>

<bondArray>

      <bond atomRefs2="a1 a2" id="a1_a2" order="S"/>

      <bond atomRefs2="a1 a3" id="a1_a3" order="S"/>

      <bond atomRefs2="a1 a4" id="a1_a4" order="S"/>

      <bond atomRefs2="a1 a5" id="a1_a5" order="S"/>

     </bondArray>

    </molecule>

   </module>

  </module>

 </module>

</module>
